# A new model for pharmacies? Insights from a quantitative study regarding the public’s perceptions

**DOI:** 10.1186/s12913-019-3987-3

**Published:** 2019-03-21

**Authors:** Verónica Policarpo, Sónia Romano, João H. C. António, Tânia Sofia Correia, Suzete Costa

**Affiliations:** 1 Instituto de Ciências Sociais, Universidade de Lisboa, Av. Prof. Aníbal de Bettencourt, 9, 1600-189 Lisbon, Portugal; 2 Centre for Health Evaluation & Research (CEFAR), Associação Nacional das Farmácias (ANF), Rua Marechal Saldanha 1, 1249-069 Lisbon, Portugal; 3 Research Centre of Public Opinion, Universidade Católica Portuguesa (CESOP), Palma de Cima, 1649-023 Lisbon, Portugal; 4 USFarmácia® Collaborative Care Project, Associação Nacional das Farmácias (ANF), Rua Marechal Saldanha 1, 1249-069 Lisbon, Portugal

**Keywords:** Community pharmacy, Satisfaction, Pharmacy services, Evaluation of pharmacy services, Portugal

## Abstract

**Background:**

Worldwide community pharmacies are shifting their role in the healthcare system from simple medication dispensers to health care providers. High levels of satisfaction with pharmacy services were found in previous studies. This study has two main goals. The primary goal is to describe the levels of satisfaction and knowledge regarding pharmacy services in Portugal. The secondary goal is to explore the perceptions and the utilisation of pharmacy services by the Portuguese. This statement includes exploring the impact of a set of variables on both perceptions and uses of pharmacies in regard to services that are currently offered as well as to new services that may be provided in the future.

**Methods:**

A face-to-face survey of closed-ended questions was applied to a nationwide representative sample of the Portuguese population in September 2015. The sample was weighted based on population distribution across regions, habitat, age and gender. Data analysis comprises descriptive statistics and Multiple Correspondence Analysis to explore different typologies of respondent’s orientation toward community pharmacy.

**Results:**

A total of 1114 interviews comprised the study. Of the respondents, 36% used the pharmacy as a first resource when seeking to treat a minor ailment, and 54% reported that they use the pharmacy as a first resource when seeking answers about medicines. Of those who visited their pharmacy at least once in the previous year, 94% were either globally satisfied or very satisfied. The level of acknowledgement of pharmacy services’ was also high among the Portuguese. Of the participants, 29% considered there could be more services available in pharmacies that are currently provided by other health care facilities. The construction of a typology of orientations towards community pharmacy practice resulted in three outcome groups: “Motivated” (63%), those with a connection to a pharmacy; “Settled” (23%), mainly those who had a pharmacy nearby; and “Demobilised” (14%), those who are weakly tied to a pharmacy.

**Conclusions:**

The vast majority of the Portuguese population has a strong positive attitude towards their community pharmacy, as expressed by the high levels of satisfaction with, and positive evaluation of, the pharmacy’s services.

**Electronic supplementary material:**

The online version of this article (10.1186/s12913-019-3987-3) contains supplementary material, which is available to authorized users.

## Background

Worldwide community pharmacies are shifting their role in the healthcare system from simple medication dispensers to health care providers [1]. With this paradigm shift, it is relevant to understand the following: what pharmacy users want more of from their pharmacies; what are their expectations regarding a new set of advanced services that pharmacies may offer in the near future and; how do such expectations relate to the level of satisfaction regarding the services that are currently being provided. This paper attempts to answer these questions, utilising quantitative data from a study conducted in Portugal in 2015, based on a nationwide statistically representative sample of the Portuguese population.

The topic is approached from the perspective of the main challenges that public health confronts in the twenty-first century, including economic crises, widening inequalities, the ageing population, increasing levels of chronic disease, migration and urbanisation, environmental damage and climate change [2]. Addressing health related matters requires the joint action of health and non-health sectors, public and private sectors and citizens [3]. Consequently, the enhancement of the role of community pharmacies in the healthcare system is a new approach that is highlighted by the political programme of the XXI Government of Portugal [4]. These issues are discussed in this paper, in the context of Portuguese community pharmacy practice, which constitutes an interesting case study to observe the transformation of the role of community pharmacies in the health care system, including the means by which pharmacy users evaluate their services and the expectations they develop towards new ones.

The Portuguese health system is organised around a National Health Service (NHS) that is primarily funded through transfers from the government budget, meaning that general taxation is the main source of funding for the NHS. The Portuguese NHS derives from the Beveridge model and is universal in coverage, general in provision and tendentially free for its users. The health care delivery system consists of a network of public and private health care providers; each is connected to the Ministry of Health and to the patients in its own way [5].

Portuguese community pharmacies are privately owned, and each has a technical director with a degree in pharmaceutical sciences [5]. By the end of 2015, there were 2936 pharmacies distributed all over the country with an average of seven full-time employees per pharmacy [6, 7]. The rural areas are the regions where the number of pharmacies per capita is higher [7], providing proximity support to this population with lower direct access to other health care providers.

The services provided by Portuguese community pharmacies include, among others, the sale of prescription-only medicines (POM) and non-prescription medicines (NPM) and several other health-related products (e.g., cosmetics and vitamins). Although pharmacists have always had involvement in providing health services, this trend has increased in recent years [8, 9]. In 2007, with recent updates, a new legislation defined the scope of services allowed in pharmacies, including: home care support, first aid, medicines administration, immunisation (vaccines not included in the National Vaccination Plan), lab tests, diagnostic and therapeutic services, disease management programmes, health campaigns and collaboration in national health education programmes [10, 11]. Therefore, as a setting for public health activities, community pharmacies present several benefits for its users. With extended business hours and no appointment needed in advance, these pharmacies can be more accessible than other health care providers [12].

Conversely, a review conducted in 2008 identified high levels of patient satisfaction with pharmacy services and highlighted the need to understand expectations and preferences of patients, exploring and matching these to the services provided [13]. Such high levels of satisfaction with the community pharmacy services have also been reported by the Portuguese population in previous studies [14]. For many years, pharmacists have been in the top 5 of the most trusted professions according to consumer surveys [15, 16]. Duarte et al. [17] showed how pharmacy clients evaluate very positively the quality of the services provided (rated as 4 or more, in a scale of 1 to 5), as well as the quality of contact and interpersonal relationship between clients and professionals. The highest score is trust (4.4), followed by personalisation (4.1) and the anticipation of needs (3.6). In their qualitative study about community pharmacy in Portugal, Cavaco et al. [18] found that the Portuguese appeared to ascribe a particular social role to the pharmacist, as part of a supportive network that contributes, among other issues, to solve problems of access to the health system. However, this role appears to be more associated to the perceived deficiencies of other community health care providers (such as doctors) than to the perceived professional duties of pharmacists. Cavaco et al^.^ [18] also observed that the interpersonal relationship of each client with the pharmacy staff was at the core of a general positive experience of the pharmacy, namely, a sense of security in relation to the adequacy and quality of dispensed products.

As such, pharmacists are considered to be knowledgeable, reliable, trustful and considerate, which is a result that has also been found in countries such as Iran [19], Canada [20], Thailand [21] and Australia [22, 23]. Moreover, the general level of satisfaction towards pharmacies is higher than towards other health care providers, such as clinical labs, private medical practices, or private hospitals. Similar results were found in Canada [20], where pharmacists were viewed positively and considered to play a central role in the health system of their provinces by a large majority of the population, mainly by women, those over 60, and regular visitors to the pharmacy. A wide majority also agreed that expanding the role of pharmacists in health care delivery would positively impact the patients’ quality of life, reduce overcrowding in emergency rooms and walk-in clinics and reduce overall health care costs [20].

While this study targets exploring similar dimensions, such as satisfaction, of the relationship between pharmacy providers and users, it also targets addressing this relationship from a different angle. Namely, this study elucidates how pharmacy users perceive the existent model of community pharmacy in Portugal, defined through the services offered, and their attitudes towards new services and therefore towards emergent new models of community pharmacy practices. In addition, based on these aspects, this study proposes a typology of community users, according to their attitudes towards both existing and emergent pharmacy models.

### Main goals and research questions

This study reports the results of a nationwide survey where the views of the Portuguese population regarding the existing pharmacy model and a proposed model to be offered in the future were explored.

Our objective is to assess the viability of possible emerging models for community pharmacy practice in Portugal. Finally, we intend to do the following: build a typology of pharmacy users towards community pharmacy practice; to characterise each cluster in terms of their composition (who are the persons that are mainly contributing to each cluster); and to identify explanatory factors for the cluster fit.

We intend to answer the question “what are the expectations of pharmacy clients in relation to a set of new services that would expand the role of pharmacies as health care providers?” In addition to this main question, a few others will be addressed: how do clients rate the importance of the services that are currently provided by the pharmacies? In addition, how satisfied are they with those services? To what extent are these levels of satisfaction related to the frequency of visits to the pharmacy and to the expectations towards enlarging their role as health care providers? The objectives of this paper are to explore possible emerging models for community pharmacy practice in Portugal and to characterise the opinions of the Portuguese population in relation to these emergent models.

## Methods

The study utilised a quantitative design, and data were collected through a survey of closed-ended questions (English translated version provided in Additional file 1). The survey was designed and conducted by CESOP (Research Centre of Public Opinion of the Catholic University of Portugal), with the collaboration of CEFAR (Centre for Health Evaluation & Research of the Portuguese Association of Pharmacies). The data were collected by 88 interviewers under the supervision of 22 field-work coordinators. A pre-test was conducted to evaluate the questionnaire understanding and feasibility. As one of the goals was to measure the perceptions of community pharmacy services in the general Portuguese population and not exclusively on pharmacies’ usual customers, a representative national sample was utilised, in accordance with a stratified multi-step method. Firstly, the localities were chosen randomly from each of the 10 strata groups obtained through combining the 5 NUTSII (Nomenclature of Territorial Units for Statistics) regions in Portugal mainland with their habitat (rural/urban). The number of localities selected from each stratum was proportional to the number of adults living in each stratum, based on the electoral register database. In Portugal, every citizen over 18 is immediately included in the electoral register. Certain residents without Portuguese nationally, namely, immigrants from outside the E.U., whom are approximately 3% of the total population, are not included in this database. Nevertheless, these individuals could yet be selected as respondent as their distribution in the country is similar to the Portuguese distribution. Secondly, for each chosen locality, the household was selected through a random route path. Thirdly, in each household, the respondents were chosen according to the method of the last/next person to have his/her birthday of 18 years old or more. Utilising a door-to-door methodology, the survey was applied face-to-face by skilled interviewers with special training for this project. A total of 1114 interviewees comprised the final sample (margin of error of approximately 3% for a 95% confidence interval). The sample was weighted based on population distribution across regions and habitat (urban/rural areas), age and gender, according to the population estimates provided by Statistics Portugal [24] (the official statistics institution in Portugal, www.ine.pt). This post-sampling weighting process allows the correction of deviations resulting from the sampling process, making the working database as near as possible to the universe under analysis.

Descriptive statistics, both univariate and multivariate, and statistical tests comprised methods of analysis to measure possible differences between groups and test the research hypothesis. After a descriptive analysis of the main variables, crosstabs enabled testing of differences according to the main characterisation variables (gender, age cohort, and region of residence), as well as other relevant variables considering the object of study (e.g., frequency of pharmacy use, or having a chronic disease). Several techniques and steps comprised multivariate exploration of data. First, Factor Component Analysis of the variables that measured the degree of importance given to pharmacy services (varimax rotation; KMO = 0.838; Bartlett’s Sphericity Test *p* < 0.001. *N* = 754) was conducted. Three components were found, and the variables with scores above 0.4 were retained to construct the new variables (α > 0.6): support with therapy and medical prescription; prevention for a healthy life; and home and other proximity services. Further cluster analysis showed that the most numerous group was the first one (56%), followed by home and other proximity services (34%) and prevention (10%). These groups were then used as input variables for a Multiple Correspondence Analysis (MCA), together with two other variables: the opinion toward the adequacy of the pharmacy services to the respondents’ needs; and how likely they were to visit the pharmacy in the future, in case it provides a particular set of new services. The analysis provided two main dimensions and suggested the organisation of responses into three groups, which were further validated through a K-Means Cluster Analysis. The groups are presented in the next section.

## Results

A total of 1114 people participated in the study. Before weighing, 64 % were women, and participants’ age ranged widely, between 18 and 95 years old (Mean 52.70, *SD* = 17.19). Participants’ distribution across the five main regions of Portugal mainland was the following: North 40%; Centre 18%; Lisbon 30%; Alentejo 6%; and Algarve 6% (Table 1). All analysis was performed after weighting the sample on the basis of the population distribution across regions and habitat (urban/rural areas), age and gender.

**Table 1:** Characteristics of survey respondents

Characteristics	Total (n=1114)			
	Non-weighted (valid)		Weighted (valid)	
	n	%	n	%
Region				
North	443	39.8	422	37.9
Centre	197	17.7	270	24.3
Lisbon	336	30.2	295	26.5
Alentejo	70	6.3	80	7.2
Algarve	68	6.1	46	4.1
Gender				
Male	394	35.5	536	48.1
Female	715	64.5	578	51.9
Community pharmacy frequency				
Visit pharmacy at least once in the last year	1055	94.7	1057	94.9
≥ 6 visits in the last six months	576	54.8	538	51.1
Age group (years)				
18-24	55	4.9	99	8.9
25-34	116	10.4	177	15.9
35-44	228	20.5	208	18.7
45-54	218	19.6	201	18
55-64	171	15.4	172	15.4
≥65	325	29.2	257	23.1
Marital status				
Has a partner (married or *de facto*)	680	62	645	58.8
Single	205	18.7	281	25.6
Widow / divorced / separated	211	19.3	172	15.6
Education				
No education	33	3	24	2.2
Primary education (4^th^ to 6^th^ year)	376	34.7	337	31.1
Primary education (9^th^ year)	171	15.8	169	15.5
Secondary education (12^th^ year)	238	21.9	254	23.4
Higher education (university)	267	24.6	301	27.8
Occupation				
Employed	551	49.7	591	53.3
Unemployed	116	10.5	115	10.3
Pensioner	329	29.7	278	25.1
Other	113	10.2	127	11.3

### Use of pharmacies for minor ailments

When the participants were asked about the first place they consult when a minor ailment arises, more than one-third of the respondents (36%) answered ‘the pharmacy’, followed by primary health centres (27%). The majority of the respondents (54%) stated the pharmacy was also the first resource they seek when they have questions about medicines. Of the participants, 17% (*n* = 185) reported “other” sources, of which 48 individuals referred to the medicine leaflet, while 42 individuals referred to the internet. The pharmacy is a frequent option for the Portuguese population: the overwhelming majority of respondents (95%, *n* = 1057) reported to have visited the pharmacy at least once over the past year. Among these respondents, approximately half (51%, *n* = 538) visit the pharmacy 6 or more times, and 81% (*n* = 850) visit at least 3 times over the past 6 months. This frequency is higher among women (*p* < 0.001), the elderly (*p* < 0.001), the retired, pensioners and widowers (*p* < 0.001).

### Satisfaction with pharmacy service

**Fig. 1:**
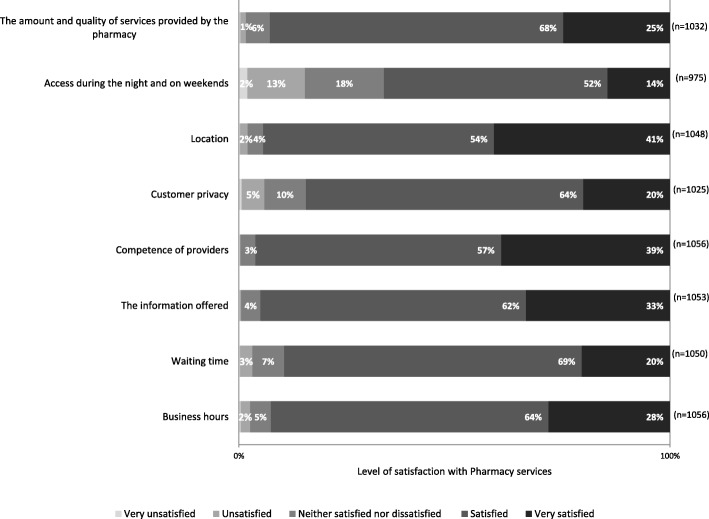
Level of satisfaction with pharmacy services

The participants appear to have a high sense of satisfaction with their Pharmacy. Among those who visited their local pharmacy at least once in the past year, the overwhelming majority reported to be either satisfied or very satisfied (94%, *n* = 995). Approximately one in four respondents (26%, *n* = 274) stated that he/she is very satisfied. The satisfaction level is also very high when the various dimensions of pharmacy services were considered separately (Fig. 1). More than 90% of respondents declared themselves satisfied or very satisfied in relation to six of the eight dimensions for which the satisfaction level was measured. Examples included the competence of providers, the information offered, the location, or the amount and quality of services provided by the Pharmacy. The dimension with the lowest score was the one related to access during the night and on weekends, followed by the customer privacy dimension. However, approximately two in every three respondents declared themselves either satisfied or very satisfied with this dimension.

### Familiarity with pharmacy services

**Fig. 2:**
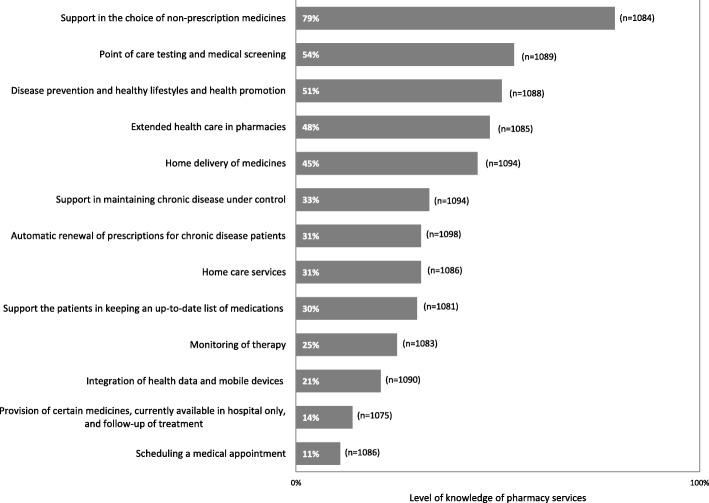
Level of knowledge of pharmacy services

**Fig. 3:**
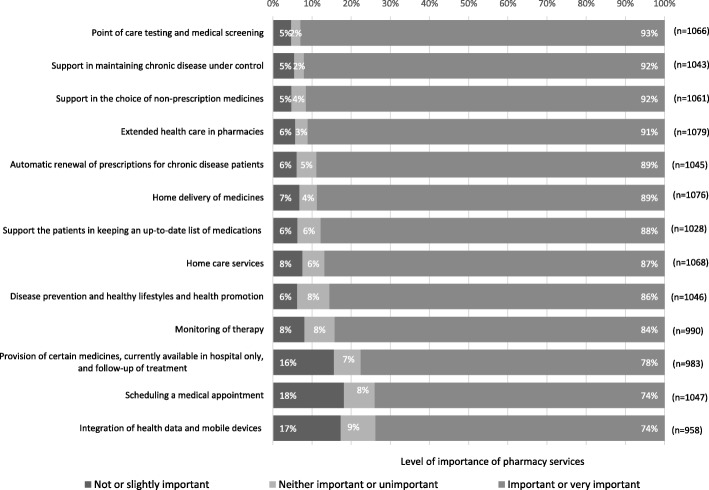
Level of importance of pharmacy services

Services provided by pharmacies appear to fit the existing needs for 69% of the respondents (*n* = 729). However, 29% (*n* = 306) of the participants believe there could be more services available in pharmacies that are currently provided in other health care facilities. Furthermore, the participants show a high level of acknowledgement of the pharmacy’s services (Fig. 2). The best-known service is the “support in the choice of non-prescription medicines”. Approximately 4 in 5 respondents (79%) reported knowing this service. Nevertheless, there are certain services that remain unknown to the majority of the participants, namely, those that are not available in Portuguese Pharmacies. The least known service is “scheduling a medical appointment” with the general practitioner via pharmacy, referred to by only one in every ten individuals (11%). Although there are services that are more familiar than others, the participants’ opinion is nearly unanimous in their importance; the vast majority of respondents rate them as either important or very important, even for services least known by respondents (Fig. 3). The most important services are “point-of-care tests and medical screening” (93% rated this service as “important” or “very important”), “support in keeping chronic disease patients under control” (92%), “support in the choice of non-prescribed medication” (92%) and “extended health care in pharmacies” (91%). When prompted to rank the five most important services, despite their current availability at the pharmacies, the respondents chose: 1) automatic renewal of prescriptions for chronic disease patients (chosen as one of the 5 most important services by 50% of the respondents and rated as the most important service by 30%); 2) support in maintaining chronic disease under control (top5: 48%; 1st: 20%); 3) home delivery of medicines (top5: 48%; 1st: 12%); 4) extended health care in pharmacies (e.g., treatment of small wounds, and first aid) (top5: 39%; 1st: 14%); and 5) provision of certain medicines exclusively dispensed at the hospital pharmacy and follow-up of treatment (top5: 38%; 1st: 14%).

### Building a typology of orientations towards community pharmacy practice

**Fig. 4:**
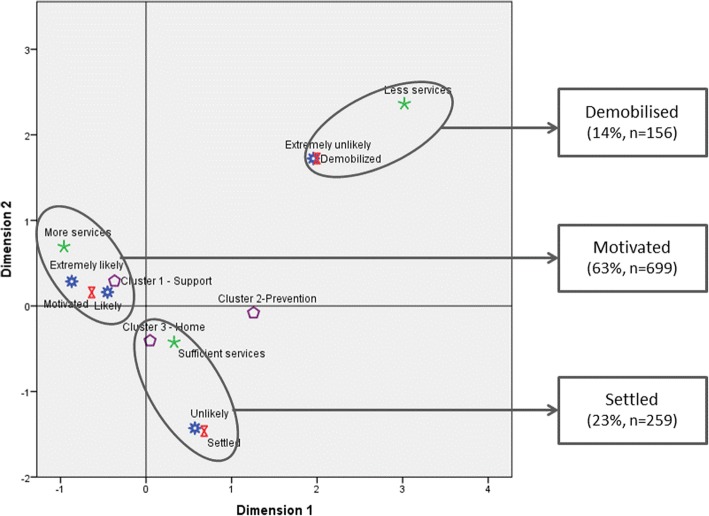
A typology of orientations towards community pharmacy practice

One of the main goals of this study was to explore possible emerging models for community pharmacy practice in Portugal and to characterise the Portuguese population in relation to those emergent models. To accomplish this goal, an MCA was conducted, having the following active variables: the opinion towards the adequacy of the pharmacy services to general needs; how likely respondents are to consider visiting the pharmacy in the future, in case it provides a particular set of new services; and a new variable classifying individuals according to how they rate the importance of pharmacy services (clusters). MCA allowed the identification of two dimensions, resulting in three outcome groups (Fig. 4). These groups are named according to the main idea that cross-cuts the variables that are most relevant to their formation. Thus, qualitatively naming the groups was a means of better capturing and communicating the main traits of each group.

#### The “Motivated”

People who reveal a connection with the Pharmacy. These people are regular customers, and we anticipate they will seek pharmacy services more often if they increase and diversify. These people want pharmacies to support chronic disease patients through extended health care, follow-up, and automatic renewal of prescriptions. The “Motivated” constitute the largest group in the sample (63%, *n* = 699). Women primarily comprise this group, as do the youngest and regular pharmacy customers.

#### The “Settled”

Mainly characterised by favouring services “coming to them” (23%). Pharmacy customers occasionally comprise this group; this group is unlikely to increase its demand for the Pharmacy. These individuals believe pharmacy services are adjusted to their needs. This group highlights the importance of services such as home delivery of medicines or provision of certain medicines exclusively dispensed at the hospital pharmacy. The “Settled” are primarily men, often older than younger, retired/pensioners, and mainly have a pharmacy nearby.

#### The “Demobilised”

This is a minority group (14% of the sample), weakly tied to the Pharmacy. Members of this group rarely visit the pharmacy and do not believe they may need to visit more often in the near future. This group is primarily composed by men, unemployed, those who are rare pharmacy customers, and people with no easy access to a pharmacy.

## Discussion

Results suggest that the vast majority of the Portuguese population has a strong positive attitude towards the community pharmacy. These results are in accordance with what other studies found in different countries [19–21, 23, 25–27], emphasising the cross-country relevance of community pharmacies as general health care providers [1, 28].

This positive attitude is three-fold. First, this attitude is expressed by the high levels of satisfaction with the pharmacy in general and with its particular services; the competence of pharmacists and the quality of services obtained the highest levels of satisfaction, confirming results from previous studies developed in Australia [22], Iran [19], Canada [20], Ireland [25], or Portugal [18]. Second, the interviewees rate as important or very important the majority of services currently provided by community pharmacies. Finally, the Portuguese also positively evaluate the possibility of pharmacies providing a new set of services in the near future. Evaluating patients’ satisfaction and attached importance with different pharmacy services plays an important role that can support and help to design and develop innovative pharmacy services to better serve patients’ needs and preferences and to maximise the professional capacity of community pharmacies at local and national levels.

These results encompass an endorsement by the Portuguese of an enlarged role of pharmacists and community pharmacies in the provision of extended health care services. A similar result was found in one studied conducted in Canada [20], which showed that 72% of Canadians considered pharmacists to be health care providers; more than half rated them as central players in the healthcare system. Similarly, previous research regarding the Portuguese context had previously highlighted that the Portuguese identify different roles for pharmacists, such as medicines supplier, advice provider and community health promoter; these are roles that were positively related to patients’ satisfaction and loyalty, explaining 50% of patients’ satisfaction and 61% of loyalty [29]. The community health promoter role equally related to both of these dimensions. However, in their qualitative study regarding the consumers’ perceptions of community pharmacy in Portugal, Cavaco et al. identified divergent understandings of the pharmacist’s role, with the main perceptions pointing to a product-supplying role and associated to low levels of knowledge and awareness of the pharmacists’ responsibilities [18]. The researchers hypothesise that such poor knowledge and low expectations can justify a reduced desire for an extended role of the pharmacist in the community. By contrast, our data suggest that the Portuguese population would welcome new services, if they are oriented to enhance better access to general health care. However, the “customer privacy” dimension needs to be addressed and potentially improved for future pharmacy service development. Our findings are most in accordance with previous studies that suggest patients’ concerns regarding privacy and confidentiality [12, 30].

As patient satisfaction emerges as a criterion to improve patients’ trust [31] and health outcomes [32], this article attempts to build a typology of pharmacy’s effective and potential users, in regard to their attitudes towards a new model of community pharmacies, composed of both old services, which are more attached to POM and NPM counselling and dispensing, and new ones, which are better described as extended health care provisions (e.g., providing POM that are currently only available through hospitals, or automatic renewal of prescriptions for patients with chronic diseases that are under control). The three groups identified suggest two different lines of action. On the one hand, pharmacies are challenged to correspond to the currently high expectations and positive attachment of their regular customers, mainly women and the youngest. The expansion of the pharmacies’ role as a general health care provider appears to fit the expectations of these “motivated” individuals, which currently appear to have a connection to the pharmacy; the challenge is to retain them in the changing dynamics of the pharmacies. On the other hand, pharmacies are challenged to address the needs of both the “settled” (23%) and the “demobilised” (14%); the first has easy access to a pharmacy, which is contrary to the second.

Targeting these groups with new and more sophisticated pharmacy services that fulfil different preferences and needs can be an opportunity to expand the patient-centred professional role of community pharmacists and to enhance the management of chronic conditions in the community with lower costs for the patients and the government [33].

A recent study estimated that the community pharmacy services provided in Portugal in the last 25 years provide a gain of 8.3% in the Quality of Life (QoL) of patients and an annual economic value of 879.6 M€, including 342.1 M€ in services other than dispensing and 448.1 M€ in avoided expenses related to health care resource use. These services included: adherence interventions; chronic disease interventions; and non-prescription selection and counselling. Potential future community pharmacy services were also estimated with an additional increase of 6.9% in QoL and an economic value of 144.8 M€ [34].

Although this study does not explore the consumers’ willingness to pay (WTP) for pharmacy services, which may dictate the feasibility of their implementation, several studies report that there are many consumers willing to pay for specific community pharmacy services. The willingness to pay appears to be sensitive to perceived utility and health improvements [35–37].

Developing and expanding the role of community pharmacists can be a means to renew the focus on preventive health care and to extend the coverage of primary health care. For example, in Finland, community pharmacists are actively involved in the treatment and prevention of major chronic diseases. In the United States, community pharmacies provide influenza immunisations in communities designated as medically underserved [38], and in England, the New Medicine Service (NMS), a complex medicine management intervention, supports patients’ adherence to newly prescribed medicines for long-term conditions [39]. Broadening the scope of community pharmacists’ may be an important policy lever that countries could pursue to provide less costly and more preventive health care and better management of long-term conditions [40], as patients show strong attitudes, connection and satisfaction with these professionals and services provided.

## Conclusion

This study explored a new approach involving an understanding of the expectations and preferences of patients towards community pharmacies, leading to an exploratory typology of (potential) pharmacy customers. One contribution of this article is that it provides insight regarding the basis of a statistically representative sample of the Portuguese population. In a face-to-face interview conducted door-to-door, 1114 respondents answered a closed-question survey that measured their attitudes in relation to the services that community pharmacies currently provide, as well as those that could be provided in the near future. This face-to-face methodology, which is known to have an advantage in obtaining quality data, also has its disadvantages, as is the case when interviewees diligently attempt to meet the (imagined) expectations of the interviewers and therefore adhere more easily to socially acceptable norms and discourses. This behaviour may have influenced the very high levels of satisfaction towards community pharmacy services and may be one of the limitations of the study. Nevertheless, this methodology of face-to-face interviewees is rated as one of the best for ensuring trust among interviewees and therefore the reliability of the information and data produced.

The results suggest that the majority of the Portuguese have a connection to a pharmacy, in accordance with the results from previous studies on community pharmacy across the world and in Portugal. The levels of satisfaction are high, and the services provided are rated as important and very important by the majority of the population. On the one hand, the importance assigned to services is associated with the knowledge that customers currently have about them (the more knowledge, the more importance). On the other hand, respondents are open to new services that have not yet been implemented, which they also perceived as important. These results suggest that there is a general agreement among the population regarding the role of pharmacies in the health system and a positive attitude toward the extension of their role as public health providers.

Additional investigations to determine consumers’ willingness to pay for different pharmacy services should be explored.

This study highlights certain future directions that can be pursued by patients, health professionals and policy makers to expand the health care role of Portuguese community pharmacies.

## Additional file

Additional file 1: Survey template. English translated version of the Portuguese survey. (PDF 349 kb)

## Abbreviations

CEFAR: Centre for Health Research & Evaluation
CESOP: Research Centre of Public Opinion of the Catholic University of Portugal
M€: Million euros
MCA: Multiple Correspondence Analysis
NHS: National Health Service
NMD: New Medicine Service
NPM: Non-prescription medicines
NUTS: Nomenclature of Territorial Units for Statistics
POM: Prescription-only medicines
QoL: Quality of live
WTP: Willingness to Pay
