# Supplementary material for: A new model for pharmacies? Insights from a quantitative study regarding the public’s perceptions
Source: BMC Health Serv Res. 2019 Mar 21;19:186. doi: 10.1186/s12913-019-3987-3 (PMC6429700; doi:10.1186/s12913-019-3987-3)
Supplement: Supplementary file 1 — Survey template. English translated version of the Portuguese survey. (PDF 349 kb) [file 12913_2019_3987_MOESM1_ESM.pdf]

Version: Definitive. Interviewee phone:______________________ **Survey nº** [| | |]

CESOP New Pharmacy Model

Date ____/09/2015 Start time ___:____ End time ___:____ Interviewer:_______________________________ [| | |]

Street ____

Good morning/afternoon/evening. My name is________ and I´m working with Centro de Sondagens da Universidade Católica in collaboration with Centro de Estudos e Avaliação em Saúde (CEFAR) from the Associação Nacional das Farmácias (ANF) in carrying out a study that aims to gather some information about the access to medicines and the Portuguese population´s preferences for a future pharmacy model. Can you please answer some questions about this subject? YOUR ANSWERS ARE ABSOLUTELY CONFIDENTIAL AND WILL BE USED ONLY FOR THIS STUDY'S PURPOSE.

ASK THE INTERVIEWEE WHICH RESIDENT IN THE HOUSE (18 YEARS OR OLDER) IS CELEBRATING THEIR BIRTHDAY NEXT

Localities (WHITHOUT ASKING)

- ☐ Alijó
- ☐ Carregosa
- ☐ Várzea, Aliviada e Folhada
- ☐ UF Braga (S. José de S. Lázaro e S. João do Souto)
- ☐ Valongo
- ☐ Espinho
- ☐ UF Antuzede e Vil de Matos
- ☐ Gafanha do Carmo
- ☐ UF Marrazes e Barosa
- ☐ Esmoriz
- ☐ Algueirão-Mem Martins
- ☐ São Domingos de Rana
- ☐ Pinhal Novo
- ☐ Odivelas
- ☐ Alcântara
- ☐ UF Romeira e Várzea
- ☐ UF Ponte de Sor, Tramaga e Vale de Açor
- ☐ Mexilhoeira Grande
- ☐ Quelfes

I - ACCESS TO PHARMACY

P1. **What is the first place you go to for advice when a minor ailment arises (e.g. headaches, dyspepsia, acute cough, influenza)?**

- ☐ Primary Health Care
- ☐ Pharmacy
- ☐ Hospital
- ☐ Hotline (Linha Saúde 24)
- ☐ Other (specify) ______________________________
- ☐ I do not usually consult anyone, anywhere
- ☐ *Don't know*

P2. **What is the first place you go to when you have any questions about medication, (e.g. doubts about dosage, indication, how is used, adverse effects)?**

- ☐ Primary Health Care
- ☐ Pharmacy
- ☐ Hospital
- ☐ Other (specify)? ______________________________
- ☐ I do not usually consult anyone anywhere
- ☐ *Don't know*

P3. **In the past year, have you visited the pharmacy?**

- ☐ Yes
- ☐ No → **go to 0**
- ☐ *Don't know* → **go to 0**

P4. **And in the last 6 months, how often did you visit the pharmacy?** (READ)

- ☐ 6 or more times
- ☐ Between 3 to 5 times
- ☐ Twice
- ☐ Once
- ☐ Never
- ☐ *Don't know*

P5. **How long does it take, on average, and using your usual means of transportation (foot, car or public transport), from your residence to the following places?** (READ)

			Usual means of transportation		
	Minutes	*Prefer not to answer*	On foot	Car	Public transport
Primary Health Care	[|]	☐	☐	☐	☐
Pharmacy	[|]	☐	☐	☐	☐
Hospital	[|]	☐	☐	☐	☐

II – SATISFACTION WITH PHARMACY

Only for those who visited the Pharmacy at least once in the last year (P3 = Yes)

If have NOT visited the Pharmacy in the last year → Go to section III

P6. **Thinking about your pharmacy (the one you visit the most), how satisfied you are with:** (READ)

	Very unsatisfied	Unsatisfied	Neither satisfied nor dissatisfied	Satisfied	Very satisfied	*Don't know*
Business hours	☐	☐	☐	☐	☐	☐
Waiting time	☐	☐	☐	☐	☐	☐
The information offered in response to your needs	☐	☐	☐	☐	☐	☐
Competence of providers	☐	☐	☐	☐	☐	☐
Customer privacy	☐	☐	☐	☐	☐	☐
The location	☐	☐	☐	☐	☐	☐
Access during the night and on weekends	☐	☐	☐	☐	☐	☐
The amount and quality of services provided by the pharmacy	☐	☐	☐	☐	☐	☐

P7. **In general, how satisfied are you with your pharmacy?** (READ)

- ☐ Very unsatisfied
- ☐ Unsatisfied
- ☐ Neither satisfied nor dissatisfied
- ☐ Satisfied
- ☐ Very satisfied
- ☐ *Don't know*

III – ACCESS TO MEDICINES

P8. **Are you a chronic disease patient or do you take medicine(s) continuously?**

☐ Yes ☐ No ☐ *Prefer not to answer*

P9. **In the last 12 months did you have to go back to the pharmacy, or go to more than one pharmacy, to get all your prescription drugs?**

☐ Yes ☐ No ☐ *Don't know*

P10. **In the last 12 months, were you, for any reason, unable to buy a medication prescribed by your doctor?**

- ☐ Yes
- ☐ No → **go to P12**
- ☐ *Don't know* → **go to P12**

P11. **(If yes) What was the most common reason not to buy the medicine(s)?** (SELECT 1 ANSWER) (READ)

- ☐ Still had the medicine / package home
- ☐ I could not find it in any pharmacy
- ☐ Had less money
- ☐ The reimbursement of the drug has decreased / ended
- ☐ I do not get along with the medicine(s)
- ☐ Other. (specify) ________________________________
- ☐ *Don't know*

IV – PHARMACY MODEL

P12. **Currently, pharmacies provide a set of services such as vaccinations, medicines counselling, point of care testing for diabetes, among others. To improve public health, in relation to the services provided by pharmacies, you consider that:** (READ; choose **1** answer)

- ☐ Pharmacies could provide more services that are currently only provided by other health care units
- ☐ The services provided currently by the pharmacies are enough to cover the population's needs
- ☐ Pharmacies could reduce the number of services provided
- ☐ *Don't know*

P13. **I will now show you a list of services that are available at the pharmacy, or could be available in the future. Regarding each one , can you please tell me if: 1) You know or have heard about it; 2) How important you rate each service (in a scale from 1 - not important to 5 - very important).** (SHOW CARD 1) (READ)

	Know / have heard of	Do not know / have not heard of	1 - Not important	2 - Slightly important	3 - Neither important or unimportan	4 - Important	5 – Very important	*Ns/Nr*
**1. Automatic renewal of prescriptions for chronic patients** (Prescription renewal for chronic patients at the Pharmacy, with no need to go to the Primary Health Care / Family Health Practice to get a prescription)	☐	☐	☐	☐	☐	☐	☐	☐
**2. Support in maintaining chronic disease under control** (Follow-up of chronic patients (e.g. diabetics, hypertensives, chronic respiratory disease, etc.) at the Pharmacy with information shared with the General Practitioner)	☐	☐	☐	☐	☐	☐	☐	☐
**3. Integration of health data and mobile devices** (Use of an integrative App where the user can download their health information (e.g. blood pressure) from various mobile devices (e.g. phones). The collected data could be evaluated together with the pharmacist and, in case of need, referred to the doctor)	☐	☐	☐	☐	☐	☐	☐	☐
**4. Monitoring of therapy** (e.g. control of therapeutic effects, adverse events, adherence)	☐	☐	☐	☐	☐	☐	☐	☐
**5. Support in the choice of non-prescription medicines** (Minor ailments advice allowing a quick and effective access to treatment)	☐	☐	☐	☐	☐	☐	☐	☐
**6. Support the patients in keeping an up-to-date list of medicines** (For the patient / doctor to compare with the prescribed medicines at hospital stay, in transition or discharge situations)	☐	☐	☐	☐	☐	☐	☐	☐
**7. Scheduling a medical appointment** (Possibility of scheduling a medical apointment in the NHS through the pharmacy according to the manifested need)	☐	☐	☐	☐	☐	☐	☐	☐
**8. Home delivery of medicines**	☐	☐	☐	☐	☐	☐	☐	☐
**9. Home care services** (Differentiated assistance to the elderly, isolated or having difficulties in their health and medication management, in partnership with the Health Center unit)	☐	☐	☐	☐	☐	☐	☐	☐
**10. Point of care testing and screening** (identification of signs and symptoms of some health conditions and appropriate referral to a doctor (e.g. point-of-care HIV testing, urinary tract infection, diabetes)	☐	☐	☐	☐	☐	☐	☐	☐
**11. Disease prevention, healthy lifestyles and health promotion** (e.g. structured programs delivered at the pharmacy integrated into the health system, such as weight loss, and smoking cessation programs)	☐	☐	☐	☐	☐	☐	☐	☐
**12. Extended health care in pharmacies** (e.g. first aid, handling of minor wounds or burns, nutrition consultation, diabetic foot care)	☐	☐	☐	☐	☐	☐	☐	☐
**13. Provision of certain medicines exclusively dispensed at the hospital pharmacy and follow-up of treatment** Access to medicines currently only available at hospital level (e.g. cancer drugs, rheumatoid arthritis, Hepatitis C), at a community pharmacy of your choice without having to go to the hospital to pick them up.	☐	☐	☐	☐	☐	☐	☐	☐

P14. **From the previous list, chose the 5 most important services and order them from 1 to 5 1 being the most important and 5 being the least** (SHOW CARD 1)

	Service		Service
**1.**	☐	**4.**	☐
**2.**	☐	**5.**	☐
**3.**	☐		

P15. **Is there any other service not mentioned in the list that you would like to see available in your pharmacy?**

☐ Yes. Which?
____
____

☐ No
☐ *Don't know*

P16. **Do you think you will probably use the pharmacy more if some of the services we have been talking about become available?** (READ)

☐ Not at all likely
☐ Unlikely
☐ Rather likely
☐ Very Likely
☐ *Don't know*

V – SOCIODEMOGRAPHIC CHARACTERISTICS

P17. **Gender** (WITHOUT ASKING)**:**

☐ Male ☐ Female

P18. **Birthday date**

☐ (Day) ☐ (Month) ☐ (Year)

P19. **Occupation** (READ)

☐ Self-employed
☐ Employee
☐ Pensioner/Retired
☐ Permanent incapacity to work
☐ Unemployed
☐ Student
☐ Homemaker
☐ Other. Which? ______________________________
☐ *Prefer not to answer*

P20. **Marital Status** (READ)

☐ Married/ or *de facto*
☐ Single
☐ widowed
☐ Divorced/ separated
☐ *Prefer not to answer*

P21. **Highest education level completed**

☐ No education
☐ Primary education (4th year)
☐ Primary education (6th year)
☐ Primary education (9th year)
☐ Secondary education (12th year)
☐ Bachelor / Graduate degree
☐ Master's degree
☐ Doctoral degree
☐ *Prefer not to answer*

P22. **How many people live here, in your house, including yourself?** ☐

P23. **On this Card 2, the following letters correspond to income brackets. Could you say which letter most closely matches your household average monthly income (after the discounts have been made):**

C	F	H	N	O	P	V	*Prefer not to answer*
☐	☐	☐	☐	☐	☐	☐	☐

AT THE END:

1. CHECK IF ALL QUESTIONS WERE ASKED AND ANSWERED
2. REQUEST PHONE NUMBER FOR EVENTUAL QUALITY CONTROL

THANK YOU
